# Bone turnover markers as predictors of hypocalcemia in patients with bone metastases receiving denosumab

**DOI:** 10.1093/jbmrpl/ziaf013

**Published:** 2025-01-21

**Authors:** Koki Tsuchiya, Yusuke Oshita, Haruka Emori, Soji Tani, Takashi Nagai, Austin Ennis, Mahoko Ishikawa, Yoshifumi Kudo, Benjamin Alman, Koji Ishikawa

**Affiliations:** Department of Orthopaedic Surgery, Showa University School of Medicine, Tokyo, 142-8666, Japan; Department of Orthopaedic Surgery, Showa University Northern Yokohama Hospital, Kanagawa, 224-0032, Japan; Department of Orthopaedic Surgery, Showa University School of Medicine, Tokyo, 142-8666, Japan; Department of Orthopaedic Surgery, Showa University Northern Yokohama Hospital, Kanagawa, 224-0032, Japan; Department of Orthopaedic Surgery, Showa University School of Medicine, Tokyo, 142-8666, Japan; Department of Rehabilitation Medicine, Showa University School of Medicine, Tokyo, 142-8666, Japan; Department of Orthopaedic Surgery, Duke University School of Medicine, Durham, NC, 27710, United States; Department of Orthopaedic Surgery, Duke University School of Medicine, Durham, NC, 27710, United States; Department of Orthopaedic Surgery, Showa University School of Medicine, Tokyo, 142-8666, Japan; Department of Orthopaedic Surgery, Duke University School of Medicine, Durham, NC, 27710, United States; Department of Orthopaedic Surgery, Showa University School of Medicine, Tokyo, 142-8666, Japan; Department of Orthopaedic Surgery, Duke University School of Medicine, Durham, NC, 27710, United States

**Keywords:** Denosumab, Hypocalcemia, Bone turnover markers, Bone metastasis

## Abstract

Denosumab treatment is effective for the prevention of skeletal-related events in patients with bone metastases. However, hypocalcemia has been recognized as a serious adverse effect of denosumab. This study aimed to identify the risk factors for hypocalcemia in patients with bone metastases. In this prospective open-label study, 35 patients with bone metastases to be treated with denosumab were recruited. During the 3 months follow-up, 9 patients (25.7%) experienced hypocalcemia. Higher bone turnover status at baseline was observed in patients with hypocalcemia than in those without hypocalcemia following denosumab administration (total-P1NP, TRACP-5b, and S-NTX: all *p* < .05). Negative correlations were observed between the lowest calcium levels and baseline bone turnover markers (BTMs) levels (total-P1NP: *r* = −0.3987; TRACP-5b: *r* = −0.3664; S-NTX: *r* = −0.3672, all *p* < .05). Multivariate logistic regression analysis revealed that patients with high BTMs (BAP > 32.1 μg/L, total P1NP > 82.3 μg/L, TRACP-5b > 866 mU/dL, S-NTX > 30.8 nmol BCE/L) had a higher risk of hypocalcemia, even after adjusting for reported risk factors, such as age, baseline calcium levels, and renal function (BAP > 32.1 μg/L: OR = 10.4; total P1NP > 82.3 μg/L: OR = 22.07; TRACP-5b > 866 mU/dL: OR = 36.5; S-NTX > 30.8 nmol BCE/L: OR = 39.74, all *p* < .05). This study shows that denosumab significantly affects serum calcium levels in patients with bone metastases who have high bone turnover status. Bone turnover markers could serve as surrogate markers to predict hypocalcemia.

## Graphical Abstract

**Graphical Abstract:**
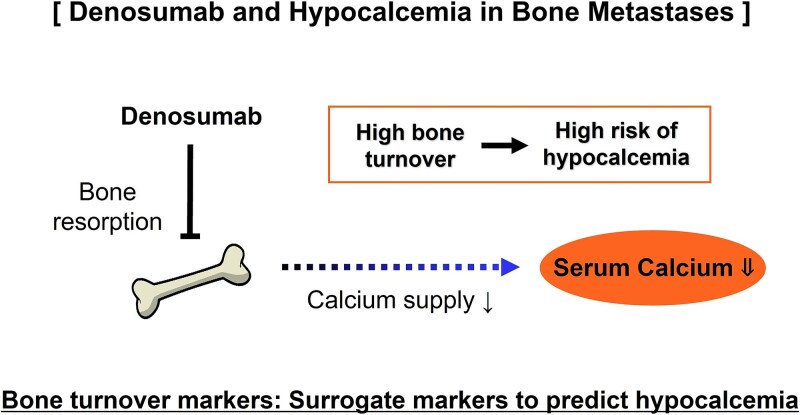

## Introduction

Bone metastases are common in advanced cancer.^1^ The incidence of bone metastases varies among cancer type. For instance, it is estimated to be 70%-80% in patients with advanced breast and prostate cancers, whereas 15%-30% in patients with lung cancer or other solid tumors.^2^ Serious complications of bone metastases can include pathological fracture, spinal cord compression, hypercalcemia, and hypocalcemia.^3^ These complications are known as skeletal-related events (SREs) and can negatively impact quality of life (QOL) for patients with bone metastases.^4^ Tumor cells interact with osteoclasts in the bone microenvironment, including a local increase in tumor-derived factors that promote osteoclastogenesis. Therefore, prevention of bone resorption is important for maintaining bone health. Denosumab (anti-RANKL antibody) has been reported to reduce the incidence of SREs in patients with bone metastases.^5^ In addition, denosumab delayed the first SRE event and even reduced the total number of SREs.^6^ Interestingly, unlike zoledronic acid, which requires renal adjustment and often triggers acute phase reactions, denosumab does not need dose changes based on renal function and generally has fewer side effects. However, hypocalcemia is a notable side effect of denosumab, with an incidence ranging from 10% to 20%.^7–11^ A recent study reported that the overall incidence of hypocalcemia grade ≥2 was higher with denosumab (12.4%) than with zoledronic acid (5.3%).^12^ Hypocalcemia can lead to life-threatening complications, such as laryngospasm and cardiac arrhythmia. For patients with bone metastases, decreased QOL caused by drug side effects are significant concerns. In patients with bone metastases, lower baseline estimated glomerular filtration rate (eGFR) is reported as a risk factor for denosumab-induced hypocalcemia.^13^^,^^14^ However, the occurrence of denosumab-induced severe hypocalcemia in patients with mild to moderate CKD (30–89 mL/min/1.73 m^2^) indicates the need for more reliable surrogate markers to predict hypocalcemia.^15^ We have reported that high bone turnover is associated with a higher risk of denosumab-induced hypocalcemia in patients with osteoporosis.^16^ Therefore, we hypothesized that bone turnover markers (BTMs) could be useful for predicting denosumab-induced hypocalcemia in patients with bone metastases. This prospective open-label study analyzes patients with bone metastases scheduled to receive denosumab, aiming to identify risk factors for hypocalcemia.

## Methods

### Study design

This study prospectively enrolled patients with bone metastases who were scheduled to start a 120 mg subcutaneous dose of denosumab between May 2016 and May 2018. All patients received oral calcium at 600 mg and alfacalcidol (activated vitamin D) at 1.0 μg as prophylactic drugs to avoid denosumab-induced hypocalcemia. The inclusion criteria for the study were cancer patients with bone metastases older than 20 yr. The exclusion criteria were: (1) adjusted baseline serum calcium concentrations below the normal range in our laboratory (8.7-10.3 mg/dL), (2) actively receiving treatment for calcium metabolism disorders, or (3) disorders such as hyperparathyroidism and malabsorption syndrome. Thirty-five patients were included in the study, with 30 completing 3 mo of follow-up. Two patients discontinued after the second injection and three after the third. The reasons for discontinuing denosumab included changing hospitals (1 patient), disease progression (1 patient), and death (3 patients).

The primary endpoint was to identify the risk factors of denosumab-induced hypocalcemia. The secondary endpoint was to evaluate the changes in serum calcium concentrations following treatment with denosumab over the treatment period. This study received approval from the Ethics Committee of Showa University (No. 1807) and was registered with the UMIN Clinical Trials Registry (UMIN000022516). Written informed consent was obtained from all participants involved in the study.

### Data collection

Data are collected from a baseline questionnaire and medical record systems, including age, weight, height, BMI, presence of visceral metastases, recent chemotherapy, and performance status.

### Blood samples

Baseline evaluations included serum levels of albumin, calcium, phosphorus, and the eGFR. The BTMs (bone turnover markers: bone-specific alkaline phosphatase [BAP], tartrate-resistant acid phosphatase 5b [TRACP-5b], total procollagen type 1 N-terminal propeptidetotal [total-P1NP], and serum N-telopeptide of type I collagen [S-NTX]) were assessed at baseline, and after 1, 2, and 3 mo following treatment. At baseline, 1 patient failed the BAP measurement, and 2 patients failed the S-NTX measurement. We defined hypocalcemia as an adjusted serum calcium concentration below 8.7 mg/dL, the lower boundary of the normal range in our central lab. The calcium level was adjusted if serum albumin levels were under 4.0 mg/dL. The severity of hypocalcemia was classified according to the National Cancer Institute’s Common Terminology Criteria for Adverse Events version 4.0. The eGFR level was estimated using the equation established by the Japanese Society of Nephrology.^17^

### Statistical analysis

Categorical variables were compared using the χ^2^ test. The Mann–Whitney U test was employed to compare groups for variables that were not normally distributed. The one-way ANOVA test is used to analyze the time course of changes in serum calcium levels. The correlations between the BTMs at baseline and nadir serum calcium were determined using Pearson’s rank correlation coefficient. Univariate and multivariate logistic regression analyses were conducted to identify risk factors for denosumab-associated hypocalcemia. A receiver operating characteristic (ROC) curve was employed to determine the optimal cutoff values for factors identified in the univariate analysis. Additionally, multiple logistic regression analyses were performed to calculate the odds ratios (ORs) for hypocalcemia induced by denosumab. Statistical analyses were conducted using Stat Flex version 7 (Artech) and GraphPad PRISM version 9 (GraphPad Software). All tests were two-tailed, and *p*-values less than .05 were considered statistically significant.

## Results

### Study population

Thirty-five patients with bone metastases who were scheduled to start a 120 mg subcutaneous dose of denosumab enrolled in this study. The clinical characteristics of the patients are shown in Table 1. The average age of patients was 71.0 yr (range 41-87 yr); 25 (74.3%) were males and 10 (25.7%) were females. In terms of primary cancer site, there were 10 (28.5%) patients with prostate, 9 (25.7%) patients with lung, 4 (11.4) patients with gastrointestinal, 2 (5.7) patients with breast, 1 (2.9) patient with multiple myeloma, and 9 (25.7%) patients with other cancer types. Renal function was normal or mildly dysfunctional (eGFR: >60 mL/min) in 27 patients (77.1%), whereas 8 patients (22.9%) had moderate kidney dysfunction (eGFR: 30-60 mL/min).

**Table 1:** Patient demographics.

**Parameters**	**Data**
**Total number of patients**	35
**Male/Female**	25/10 (71.4/28.6)
**Age, mean ± SD (yr)**	71.2 ± 11.0
**BMI, mean ± SD**	21.5 ± 4.0
**Primary cancer site**	
**Prostate**	10 (28.5)
**Lung**	9 (25.7)
**Gastrointestinal**	4 (11.4)
**Breast**	2 (5.7)
**Multiple myeloma**	1 (2.9)
**Others**	9 (25.7)
**Renal function eGFR (mL/min/1.73 m2)**	
**>60**	27 (77.1)
**30-60**	8 (22.9)
**<30**	0 (0)
**Side effect**	
**Bone pain**	14 (40.0)
**Back pain**	11 (31.4)
**Constipation**	2 (5.7)
**Arthralgia**	2 (5.7)
**Fatigue**	1 (5.7)
**Nausea**	1 (2.9)
**Asthenia**	1 (2.9)
**Vomiting**	1 (2.9)

Abbreviations: BMI: Body Mass Index, eGFR: estimated glomerular filtration rate.

### Serum calcium dynamics following denosumabadministration

**Figure 1:**
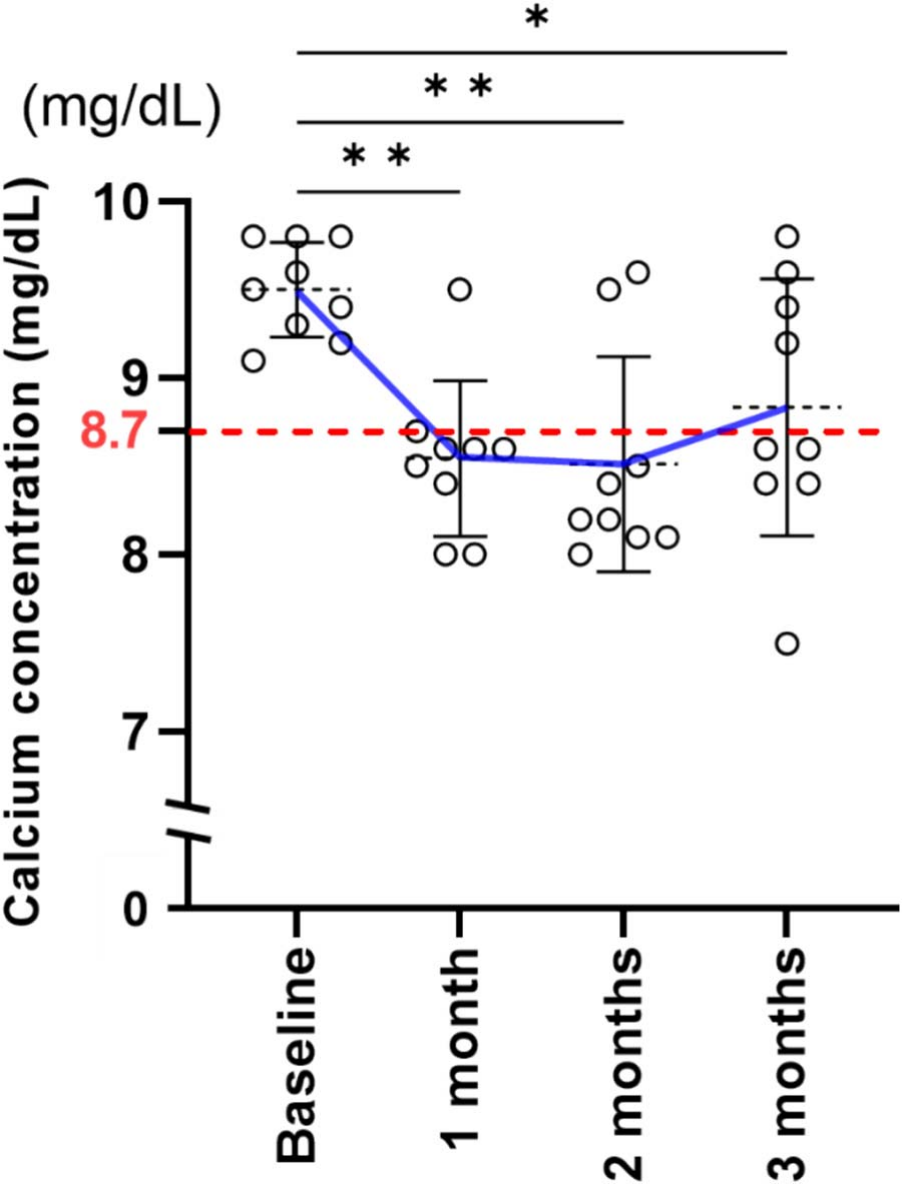
Changes in serum calcium levels over time in patients with hypocalcemia. Mean ± SD, *n* = 9, ^*^ < 0.05, ^**^ < 0.01.

Denosumab treatment effectively suppresses BTMs over time (Figure S1). None of the patients had hypocalcemia at baseline, but 9 (25.7%) developed hypocalcemia following the administration of a 120 mg subcutaneous dose of denosumab. As per the Common Terminology Criteria for Adverse Events version 4.0, 8 (22.9%) patients had grade 1 hypocalcemia and 1 (2.9%) patient had grade 2 hypocalcemia (Table 2). In patients with hypocalcemia, serum calcium levels were lowest at 1 or 2 mo, except for 1 patient (Figure 1). All patients with hypocalcemia received temporary oral calcium treatment (1000-3000 mg/daily) and were asymptomatic. Asymptomatic hypercalcemia, defined as serum calcium levels above 10.3 mg/dL (exceeding the normal range in our laboratory), was observed at least once in 5 patients (13.9%).

**Table 2:** Distribution of hypocalcemia grade.

**Grades of hypocalcemia**	**N (%)**
**All grades**	9 (25.7)
**Grade 1**	8 (22.9)
**Grade 2**	1 (2.9)
**Grade 3**	0 (0)
**Grade 4**	0 (0)

### Comparison of clinical parameters between hypocalcemia and non-hypocalcemia groups

Baseline characteristics of patients in the hypocalcemia and non-hypocalcemia groups are compared in Table 3. No significant differences were observed between the two groups, except BTMs. BTMs such as total-P1NP, TRACP-5b, and S-NTX are higher in group with hypocalcemia (total P1NP, TRACP-5b, S-NTX: all *p* < .05). This suggests that higher bone turnover is associated with an elevated risk of hypocalcemia following denosumab treatment.

**Table 3:** Baseline characteristics between the hypocalcemia and non-hypocalcemia group.

**Summary of the Parameters**	**Incidence of any grade hypocalcemia (n = 9)**	**No incidence of hypocalcemia (n = 26)**	***p*-value**
**Age (yr)**	68.0 (60.0/73.3)	76.5 (67.0/82.0)	0.100
**BMI (g/cm^2^)**	21.6 (19.2/24.4)	21.4 (18.6/25.3)	0.734
**Hb (g/dL)**	12.7 (12.0/13.4)	12.8 (10.9/14.4)	0.880
**eGFR (mL/min/1.73 m2)**	73.6 (69.3/79.7)	74.4 (59.3/84.8)	0.836
**eGFR categories: 60>, n (%)**	8 (88.9)	19 (73.1)	0.330
**eGFR categories: 30-60, n (%)**	1 (11.1)	7 (26.9)	0.330
**Ca (mg/dL)**	9.5 (9.3/9.8)	9.6 (9.3/9.8)	0.530
**Mg (mg/dL)**	2.3 (2.2/2.4)	2.3 (2.2/2.5)	0.829
**ALP (U/L)**	491.1 (313.0/727.8)	274.8 (188.0/335.0)	0.143
**BAP (μg/L)**	33.8 (14.0/61.5)	20.3 (13.2/29.8)	0.178
**total-P1NP (μg/L)**	125.9 (99.3/384.5)	67.7 (47.5/120.1)	0.016
**TRACP-5b (μU/dL)**	1338.0 (573.0 / 2099.3)	555.0 (382.0/728.0)	0.0496
**Serum NTX (nmol BCE/L)**	33.2 (25.4 / 66.6)	21.9 (18.3/28.6)	0.022
**Intact PTH (pg/mL)**	45.0 (25.0/50.3)	37.0 (29.5/59.3)	0.740
**25(OH)D (ng/mL)**	13.8 (9.4/13.8)	12.5 (10.4/16.1)	0.829
**Presence of bone metastasis**			
**None, n (%)**	0 (0)	0 (0)	–
**One place, n (%)**	3 (33.3)	10 (38.5)	0.999
**Two or more, n (%)**	6 (66.7)	16 (61.5)	0.999
**Presence of visceral metastasis, n (%)**	2 (22.2)	14 (53.8)	0.135
**Recent chemotherapy, n (%)**	3 (30)	14 (53.8)	0.443
**EPOG PS**			
**0, n (%)**	4 (44.4)	11 (42.3)	0.999
**1, n (%)**	3 (33.3)	3 (11.5)	0.630
**2, n (%)**	1 (11.1)	9 (34.6)	0.235
**3, n (%)**	0 (0)	2 (7.8)	0.607
**4, n (%)**	1 (11.1)	1 (3.8)	0.999
**Primary cancer site**			
**Prostate, n (%)**	3 (33.3)	7 (26.9)	0.135
**Lung, n (%)**	1 (11.1)	8 (30.8)	0.245
**Gastrointestinal, n (%)**	1 (11.1)	3 (11.5)	0.972
**Breast, n (%)**	0 (33.3)	2 (7.7)	0.392
**Multiple myeloma, n (%)**	0 (33.3)	1 (3.8)	0.551
**Others, n (%)**	1 (11.1)	8 (30.8)	0.245

Abbreviations: BMI: Body Mass Index, Hb: Hemoglobin, Ca: Calcium, Mg: Magnesium, eGFR: estimated glomerular filtration rate, ALP: Alkaline Phosphatase, BAP: Bone-Specific Alkaline Phosphatase, TRACP-5b: Tartrate-resistant acid phosphatase 5b, total-P1NP: total N-terminal propeptide of type I procollagen, Serum NTX: N-telopeptide of Type I Collagen, Intact PTH: Intact Parathyroid Hormone, 25(OH)D: 25-Hydroxyvitamin D, EPOG PS: Eastern Cooperative Oncology Group Performance Status.

### Correlations between baseline BTMs and serum calcium concentration

**Figure 2:**
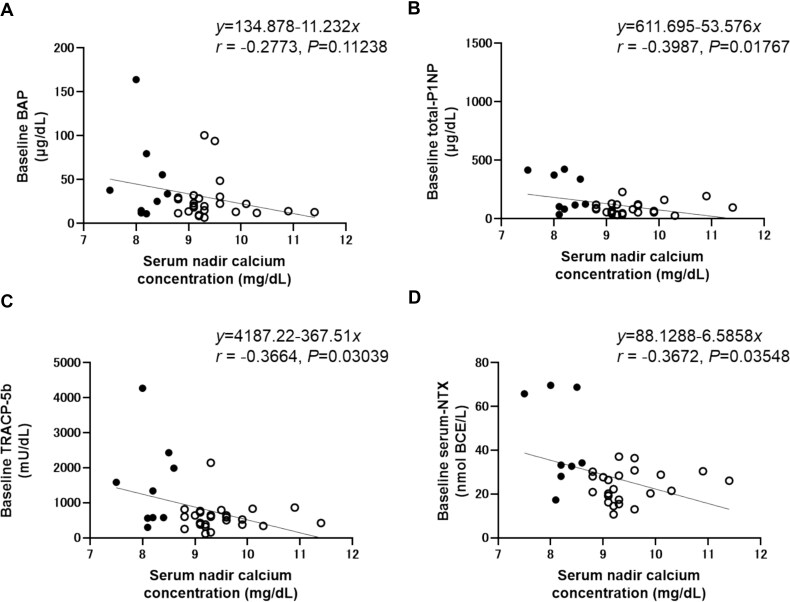
Correlations between baseline BTMs and serum nadir calcium concentration. Solid circles; patients with hypocalcemia, open circles; patients without hypocalcemia. (A) BAP, Bone-specific alkaline phosphatase, (B) Total-P1NP, total N-terminal propeptide of type I procollagen, (C) TRACP-5b, tartrate-resistant acid phosphatase 5b, and (D) NTX, N-telopeptide of type I collagen.

**Figure 3:**
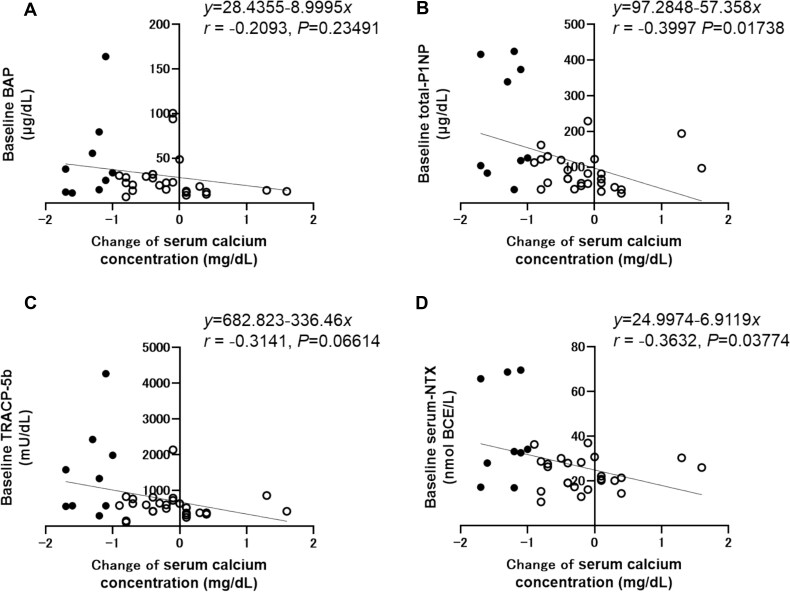
Correlations between baseline BTMs and change of serum calcium concentration. Solid circles; patients with hypocalcemia, open circles; patients without hypocalcemia. (A) BAP, bone-specific alkaline phosphatase, (B) Total-P1NP, total N-terminal propeptide of type I procollagen, (C) TRACP-5b, tartrate-resistant acid phosphatase 5b, and (D) NTX, N-telopeptide of type I collagen.

Baseline BTMs exhibited a negative correlation with the serum calcium concentration nadir [total P1NP (*r* = −0.3987, *p* = .01767), TRACP-5b (*r* = −0.3664, *p* = .03039), and S-NTX (*r* = −0.3672, *p* = .03548)] (Figure 2). Furthermore, negative correlations were observed between the change in serum calcium concentration from baseline to nadir and baseline BTMs [total P1NP (*r* = −0.3997, *p* = .01738), TRACP-5b (*r* = −0.3141, *p* = .06614), and S-NTX (*r* = −0.3632, *p* = .03774)] (Figure 3).

### Identifying hypocalcemia predictors through univariate and ROC curve analysis

Univariate logistic regression analysis identified significant associations between the incidence of hypocalcemia and levels of total P1NP, TRACP-5b, and S-NTX. To assess the predictive capacity of these BTMs for denosumab-induced hypocalcemia, the areas under the ROC curves (AUCs) were calculated based on the results of the univariate logistic regression analyses (Figure S2). The ROC curve analysis demonstrated that a baseline total P1NP level greater than 82.3 μg/L was the most robust predictor of hypocalcemia, with an AUC of 0.7735. At this cutoff value, the sensitivity for predicting hypocalcemia was 88.9%, while the specificity was 61.5%. Similarly, baseline TRACP-5b levels exceeding 866 mU/dL (AUC = 0.7222) and baseline S-NTX levels above 30.8 nmol BCE/L (AUC = 0.7485) were also identified as effective predictors of hypocalcemia.

### BTMs were identified as useful predictors of hypocalcemia through multivariate logistic regression analysis

The multivariate logistic regression analysis revealed that all BTMs were independent predictors of denosumab-induced hypocalcemia. Additionally, these significant odds ratios persisted even after adjusting for age, baseline serum calcium levels and eGFR, which is recognized as a risk factor for hypocalcemia (BAP > 32.1 μg/L: OR = 10.4, 95% CI: 1.360-79.43, *p* < .05; total P1NP > 82.3 μg/L: OR = 22.07, 95% CI: 1.757-277.3, *p* < .05; TRACP-5b > 866 mU/dL: OR = 36.57, 95% CI: 2.810-475.83, *p* < .01; S-NTX > 30.8 nmol BCE/L: OR = 39.74, 95% CI: 3.388-466.1, *p* < .01) (Table 4).

**Table 4:** Multivariate logistic regression analysis for hypocalcemia.

**Factor**	**OR (95% CI)**	***p*-value**	**Adjusted OR** ^*^ **(95% CI)**	***p*-value**
**BAP (32.1≦)**	1 (reference)	–	1 (reference)	–
**BAP (32.1>)**	9.17 (1.539-54.59)	0.0149	10.4 (1.360-79.43)	0.0241
**total-P1NP (82.3≦)**	1 (reference)	–	1 (reference)	–
**total-P1NP (82.3>)**	10.9 (1.185-100.41)	0.0349	22.07 (1.757-277.3)	0.0166
**TRACP-5b (866≦)**	1 (reference)	–	1 (reference)	–
**TRACP-5b (866>)**	31.25 (2.856-341.9)	0.0048	36.57 (2.810-475.83)	0.0060
**Serum NTX (30.8≦)**	1 (reference)	–	1 (reference)	–
**Serum NTX (30.8>)**	22.0 (2.965-162.2)	0.0043	39.74 (3.388-466.1)	0.0034

Abbreviations: BAP: Bone-Specific Alkaline Phosphatase, total-P1MP: total N-terminal propeptide of type I procollagen, TRACP-5b: Tartrate-resistant acid phosphatase 5b, NTX: N-telopeptide of Type I Collagen.

Adjusted OR^*^: The odds ratio (OR) was calculated after adjustment for age, baseline calcium (Ca), and eGFR.

## Discussion

Denosumab is widely used as a primary bone-protective agent. Given subcutaneously, denosumab is known for its high safety profile, having no impact on renal function and no association with acute phase reactions, unlike other anti-bone resorptive agents such as bisphosphonates.^18–21^ However, hypocalcemia is frequently observed with denosumab use. We have reported that high baseline bone turnover status in patients with osteoporosis, as evidenced by several elevated BTMs, is associated with a higher risk of denosumab-induced hypocalcemia.^16^ Denosumab is administered at 60 mg every 6 mo for osteoporosis and at 120 mg monthly for bone metastases. Despite differences in the bone microenvironment and dosage, denosumab suppresses bone resorption in both cases through a similar mechanism by targeting the interaction between RANK and RANKL. Hypocalcemia is significantly more common for bone metastases (5%-37%) than for osteoporosis (1.7%), underscoring the importance of our findings.^22^

The incidence of hypocalcemia in this study (25.7%) appears to be higher than those previously reported. This may be due to an apparently higher lower limit of normal serum calcium level at our institution (8.7 mg/dL) than those (8.4-8.5 mg/dL) reported elsewhere to define hypocalcemia.^6^^,^^7^ Although decreases in serum calcium levels are usually transient and asymptomatic, some patients have developed severe hypocalcemia, requiring intravenous calcium and hospitalization.^23^^,^^24^ All patients with hypocalcemia in this study received oral calcium (1000-3000 mg/daily) and remained asymptomatic, demonstrating the effectiveness of timely treatment and the use of prophylactic medication (oral calcium: 600 mg/day and activated vitamin D: 1.0 μg/day).

In contrast to our findings, previous studies that included patients with severe renal dysfunction, including those on dialysis, reported an increased risk of hypocalcemia in patients with CKD or vitamin D deficiency.^13^^,^^25^ Notably, these studies did not routinely include prophylactic supplementation in their protocols. While our study excluded patients with severe renal failure (<30 mL/min/1.73 m^2^), 11.1% in the hypocalcemia group had mild renal dysfunction, compared to 30.8% in the non-hypocalcemia group. These results suggest that mild renal dysfunction and vitamin D deficiency are not significant risk factors under this prophylactic regimen.

A stronger correlation with serum calcium levels was observed for total P1NP compared to BAP, although both are bone formation markers. This difference might be attributed to several practical advantages of total-P1NP, including its low diurnal variability, stability at room temperature, and low intraindividual variability.

Importantly, multivariate logistic regression analysis revealed that higher baseline BTMs were significant independent risk factors for hypocalcemia even after adjusting by reported risk factors such as baseline calcium levels and renal function.^13^^,^^14^ Therefore, these results reinforce the predictive value of BTMs for hypocalcemia in patients receiving denosumab, advocating for further study.

The incidence of hypocalcemia was higher with denosumab than with zoledronic acid, likely due to the strong and rapid effect of denosumab in inhibiting osteoclastic activity by binding to RANKL.^12^ This suggests a rapid reduction in bone turnover, thereby potentially lessening the release of calcium into the circulation.^12^ Patients who rely on high bone turnover to maintain normal serum calcium levels, similar to those with hungry bone syndrome, may be sensitive to hypocalcemia following denosumab treatment. The sudden shift in calcium dynamics causes a significant decrease in serum calcium levels, which can lead to severer symptoms of hypocalcemia.

The present study has several limitations. First, the number of patients was not large. Although the number of subjects is slightly fewer than our target sample size, we successfully detected predictive markers for denosumab-induced hypocalcemia. Second, we did not assess the effect of severe renal impairment, as noted in a recent report.^26^ However, we evaluated the effects of mild renal impairment and found that the impact is partial. Finally, we could not evaluate the oral calcium intake in daily life.

Our study suggests that denosumab has a notable impact on serum calcium levels in patients with elevated bone turnover. The absence of symptomatic hypocalcemia observed in this study likely reflects the effectiveness of prophylactic medications and diligent calcium monitoring, which prevented serious complications. These findings underscore the critical importance of careful monitoring of serum calcium levels, even when supplementation is provided, particularly in patients with high BTMs.

## Supplementary Material

250310-Supplymental_materials-JBMR_PLUS_final_ziaf013

## Data Availability

The data underlying this article will be shared on reasonable request to the corresponding author.
