# Supplementary material for: Bone turnover markers as predictors of hypocalcemia in patients with bone metastases receiving denosumab
Source: JBMR Plus. 2025 Jan 21;9(4):ziaf013. doi: 10.1093/jbmrpl/ziaf013 (PMC11911062; doi:10.1093/jbmrpl/ziaf013)
Supplement: 250310-Supplymental_materials-JBMR_PLUS_final_ziaf013 [file 250310-supplymental_materials-jbmr_plus_final_ziaf013.docx]

**Supplemental materials**

**Bone turnover markers as predictors of hypocalcemia in patients with bone metastases receiving denosumab**

Koki Tsuchiya^a^, Yusuke Oshita^b^, Haruka Emori^a, b^, Soji Tani^a^,

Takashi Nagai^c^, Austin Ennis^d^, Mahoko Ishikawa^d^, Yoshifumi Kudo^a^,

Benjamin Alman^d^, Koji Ishikawa*^a, d^

a. Department of Orthopaedic Surgery, Showa University School of Medicine, Tokyo, Japan

b. Department of Orthopaedic Surgery, Showa University Northern Yokohama Hospital, Kanagawa, Japan

c. Department of Rehabilitation Medicine, Showa University School of Medicine, Tokyo, Japan

d. Department of Orthopaedic Surgery, Duke University School of Medicine, Durham, NC, United States

*Corresponding author: Koji Ishikawa, Department of Orthopaedic Surgery, Duke University School of Medicine, 308 Research Drive, Durham, NC, United States (✉ koji.ishikawa@duke.edu).

Tel: (919) 681-1797

Email: koji.ishikawa@duke.edu

**Supporting Information includes:**

Supplemental Figure1 and Supplemental Figure2

**
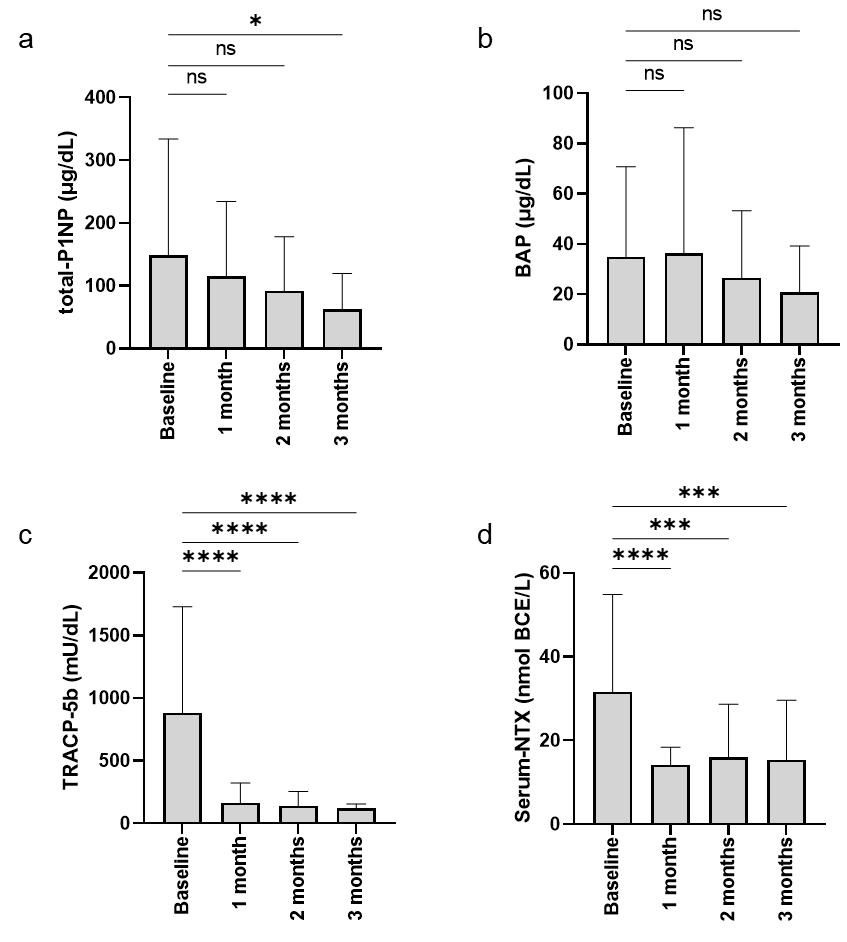
Supplemental Figure 1. Changes in BTMs over time among all patients**

Data are presented as mean ± SD. (a). BAP: Bone-Specific Alkaline Phosphatase, (b). total-P1NP: total N-terminal propeptide of type I procollagen, (c). TRACP-5b: Tartrate-resistant acid phosphatase 5b, (d). NTX: N-telopeptide of Type I Collagen

[Interval of injections: Baseline to 2nd injection; 28.9 ± 2.5 (days), 2nd to 3rd injection; 29.4±3.0 (days), 3rd to 4th injection; 29.3±3.4 (days)]


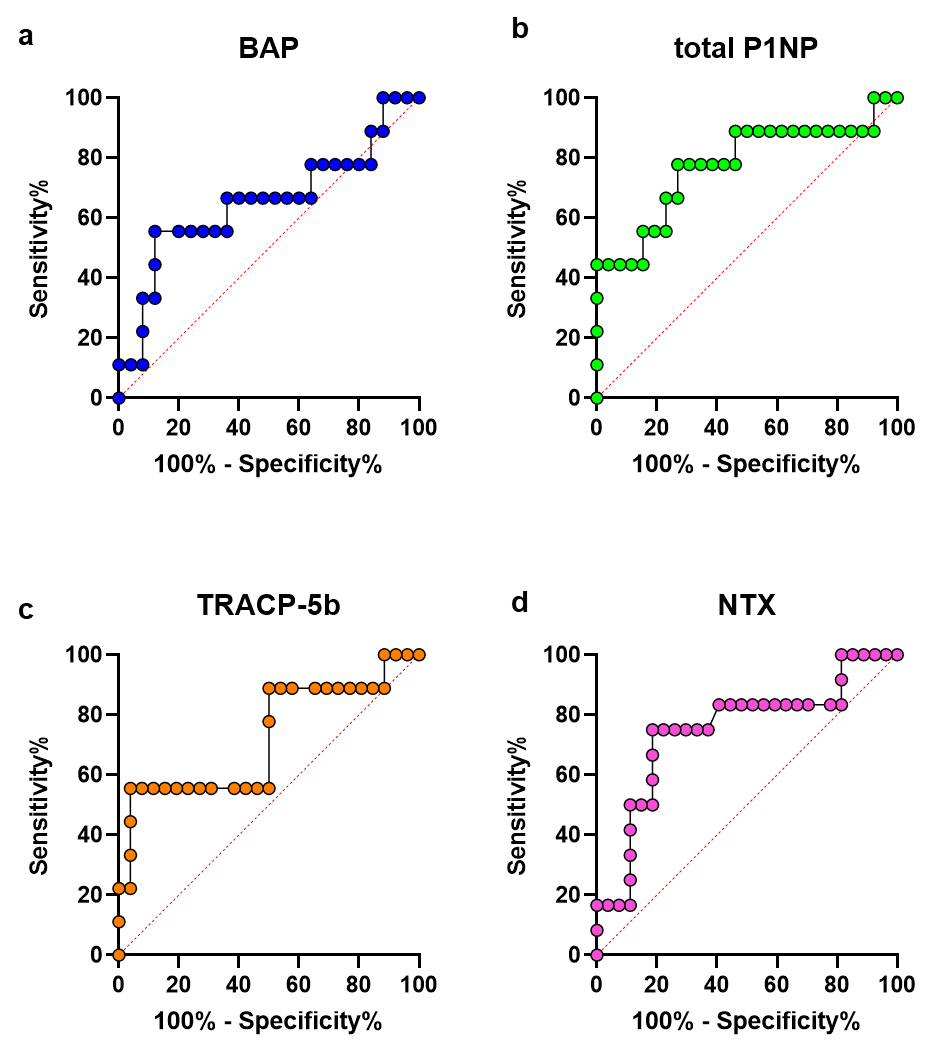
**Supplemental Figure 2. ROC curve of bone turnover markers to predict hypocalcemia**

(a). BAP: Bone-Specific Alkaline Phosphatase (b). total-P1NP: total N-terminal propeptide of type I procollagen (c). TRACP-5b: Tartrate-resistant acid phosphatase 5b (d). NTX: N-telopeptide of Type I Collagen
